# A National Resource Sharing Program Is Feasible Even in Challenging Lung Recoveries

**DOI:** 10.1016/j.atssr.2023.05.021

**Published:** 2023-06-15

**Authors:** Joel C. Boudreaux, Marian Urban, David B. Berkheim, Bronwyn Small, Heather M. Strah, Aleem Siddique

**Affiliations:** 1 College of Medicine, University of Nebraska Medical Center, Omaha, Nebraska; 2 Division of Cardiothoracic Surgery, Department of Surgery, University of Nebraska Medical Center, Omaha, Nebraska; 3 Division of Pulmonary, Critical Care, and Sleep Medicine, Department of Internal Medicine, Ohio State University, Columbus, Ohio; 4 Division of Pulmonary, Critical Care, and Sleep Medicine, Department of Internal Medicine, University of Nebraska Medical Center, Omaha, Nebraska

## Abstract

**Background:**

Mobile ex vivo lung perfusion (mEVLP) allows transportation of lung allografts while maintaining ventilation and perfusion and has demonstrated safety and efficacy with the potential to expand organ utilization. A nationwide organ recovery service has been implemented to provide surgical expertise for recovery alongside mEVLP transportation services.

**Methods:**

We reviewed patients at our institution who underwent lung transplantation with donor lungs procured with this program. Donor and recipient characteristics, procurement details, and outcomes were collected and descriptively analyzed. Individual patient consent was obtained.

**Results:**

Three patients underwent bilateral lung transplantation and 1 patient received a unilateral lung transplant with allografts procured by the recovery service. Three of the grafts were recovered from donation after circulatory death (DCD) donors and 1 from a brain-dead donor. One DCD donor was particularly challenging, requiring resternotomy. All allografts recovered were of acceptable quality and ultimately used for transplantation. The incidence of adverse events was low, and all patients survived to discharge.

**Conclusions:**

A novel national mEVLP procurement service is safe and effective even in challenging procurements. Such a program may be beneficial to increase organ utilization through expanded use of lung donors in general and DCD donors in particular. Further study is warranted for the impact on transplant program resources, organ utilization, and outcomes.

## Visual Abstract

**Figure:**
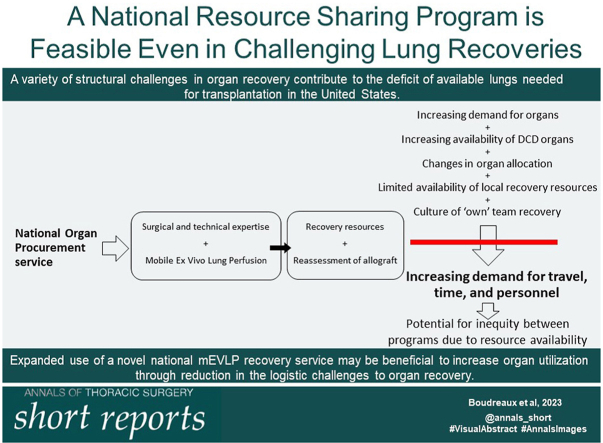

**In Short**

▪ A national system for sharing lung recovery resources is feasible and safe.
▪ Local team recovery in conjunction with mobile ex situ lung perfusion has the potential to improve the allocation of donor lungs for transplantation.

The gap between demand for organs and transplants received is well documented in lung transplantation.^1^ Mobile ex vivo lung perfusion (mEVLP) allows the transportation of allografts while maintaining ventilation and perfusion and has the potential to expand organ utilization.^2^ However, structural and logistical challenges prevent the widespread adoption of this technology. The use of non–implant center teams to recover lungs in combination with mEVLP technology has the potential to increase donor lung utilization. Herein, we discuss our experience with a novel national resource-sharing service, the National Organ Care System Program (NOP; TransMedics Inc), allowing procurement and transportation of donor lungs by local recovery teams using mEVLP.

## Patients and Methods

We retrospectively reviewed patients at our institution who underwent lung transplantation with donor lungs procured by the NOP program between September 2021 and August 2022. The program provides the technical services and expertise of a procurement surgeon and transport using EVLP on the Organ Care System device. The technique for lung recovery and use of the Organ Care System have been previously described.^3^ The primary indication for which our institution uses the program is for geographically distant donation after circulatory death (DCD) donors, in whom there is a low likelihood of progression to asystole within an acceptable time period (120 minutes of withdrawal of life support). We describe the transplantation results using quantitative and descriptive terminology. The primary outcome of interest was patient survival to 30 days. Secondary outcomes of interest related to safety included early graft function with primary graft dysfunction at 72 hours, acute rejection, respiratory failure, major pulmonary infection, and bronchial anastomotic complications. Given the small number of transplants, comparative statistics were not feasible. Individual patient consent was obtained from the patients reviewed in this study.

## Results

### Donor, Recovery, and Recipient Characteristics

Donor and recipient details are provided in Table 1. Three recipients underwent bilateral lung transplantation and one patient received a unilateral lung transplant. Three received lungs after DCD and 1 after brain death (donation after brain death). All recipients had restrictive lung physiology with a diagnosis of interstitial lung disease, and 3 recipients were 65 years of age or older.

**Table 1:** Characteristics of Recipients and Donors

Case	1	2	3	4
Recipient				
Age, y	66	70	65	62
Sex	Female	Male	Male	Female
Cause of lung failure	ILD	ILD	ILD	ILD
Lung allocation score	66.7445829	36.5879	35.0897	34.4934
Donor				
Age, y	43	63	58	60
Sex	Male	Female	Female	Female
Diabetes mellitus	No	No	No	Yes
Current smoker	No	No	No	No
Smoking history (>20 pack-years)	No	No	No	Yes
Prior sternotomy	Yes	No	No	No
Cause of death	CVA	CVA	CVA	CVA/DKA
DCD donor	Yes	Yes	Yes	No
P/F ratio	488	330	511	554
Donor neurologic examination^a^	Brainstem reflexes intact	Overbreathing vent, cough intact, posturing	Cough intact, overbreathing vent; Glasgow Coma Scale score, 9	Brain dead
Chest radiography findings	Clear	Right lung clear	Clear	Clear
Indication for NOP use	Distance, perceived low likelihood of progression to death within 120 minutes	Distance, perceived low likelihood of progression to death within 120 minutes	Distance, perceived low likelihood of progression to death within 120 minutes	Distance, surgeon availability

CVA, cerebrovascular accident; DCD, donation after circulatory death; DKA, diabetic ketoacidosis; ILD, interstitial lung disease; NOP, National Organ Care System Program.

a As reported by the donor organ procurement organization.

The rationale for using the NOP service was primarily related to logistics. In the 3 DCD cases, a combination of the long distance between donor and recipient and a perceived low likelihood of progression to asystole prompted mobilization of the service. One of these (case 3) was thought highly unlikely to progress to asystole in an acceptable time; no other solid organ transplant surgical team attended. In the fourth case, a lack of implant center procurement surgeon availability, combined with geographic distance, contributed to use of the recovery service. Notably, one DCD recovery (case 1) involved the additional surgical and technical challenge of resternotomy in a donor who had a septal defect repaired as a child. Three donors had ages >55 years. All donors had acceptable P/F ratios except for case 2, the donor in whom the right lung was used for a single transplant because there were infiltrates on the contralateral side. In all cases, lungs were transported with mEVLP and were deemed of acceptable quality for eventual transplantation.

### Outcomes

The incidence of graft-related serious adverse events was low (Table 2). One patient (case 2) experienced acute rejection, which was successfully treated with corticosteroids. Primary graft dysfunction grade 3 at 72 hours occurred in 1 of the 4 patients. All recipients survived to hospital discharge and are alive at the latest follow-up. Adverse events were not directly attributable to the recovery resources used.

**Table 2:** Transplantation Details and Recipient Outcomes

Case	1	2	3	4
Distance (donor to recipient hospital), miles	616	420	1358	560
Time to asystole,^a^ min	33	14	20	N/A
Laterality	Bilateral	Right	Bilateral	Bilateral
Right lung total ischemia time,^b^ min	234	218	153	181
Left lung total ischemia time,^b^ min	199	N/A	150	318
Other organs recovered	Kidneys	Kidneys	None	Liver, kidneys
Other	Short right lung venous cuff, no patch required	None	None	Short right lung venous cuff, no patch required
Primary graft dysfunction at 72 hours	3	1	2	0
Acute rejection^c^	No	Yes	No	No
Respiratory failure^c^	No	No	No	No
Bronchial anastomotic complication^c^	No	No	Yes	No
Major pulmonary infection^c^	No	No	No	No
30-day survival	Yes	Yes	Yes	Yes
Current status	Alive, ambulatory	Alive, ambulatory	Alive, ambulatory	Alive, ambulatory

N/A, not applicable.

a Time from withdrawal of life support to asystole.

b Cumulative time from donor asystole to reperfusion minus ex vivo perfusion time.

c Lung graft–related serious adverse events within 30 days.

## Comment

This limited clinical series illustrates the safety and feasibility of a novel national resource-sharing service for lung procurements with mEVLP. The series includes the use of the recovery service in an especially challenging donor requiring resternotomy. The presence of a previous sternotomy in a donor is a relative contraindication in thoracic transplantation because of the added surgical complexity; lungs are rarely recovered from these donors but were successfully recovered here.

In the same 1-year period, our institution's team attended 9 lung offers. One of these was DCD; all resulted in transplants, no recovery-related injury was noted, and 1 recipient died within 30 days. In addition, during this period, we requested the NOP team to attend 2 lung offers that did not result in organ recovery. One was DCD, in which the potential donor did not arrest within 90 minutes; the other was donation after brain death in which the organ was declined at the donor hospital because of functional deterioration.

Utilization of donor lungs, particularly those from DCD donors, remains low in the United States and lags behind use of other solid organs.^4^ Recent policy changes exacerbate logistic challenges to donor organ utilization as they have increased the geographic sharing of organs, travel time, distance, and cost.^5^

Ex situ lung perfusion has demonstrated safety and efficacy and has the potential to expand organ utilization.^2^ However, logistic challenges, including initial resource investment, training, and use of additional personnel, and cost presently limit its widespread use.

Sharing local resources through a program like NOP has the potential to reduce the logistic burden and to decrease geographic barriers to organ allocation. Examination of the Scientific Registry of Transplant Recipients database reveals no significant detriment to outcomes when a “different team” is used to recover lung allografts.^6^ However, the use of such teams has decreased over time, suggesting a lack of confidence in allowing unfamiliar teams to recover thoracic organs, possibly because of concerns about expertise or committing to organ acceptance on the basis of another team’s evaluation.^6^ The NOP service may reduce concerns about recovery by alternative procurement teams through ensuring the training and expertise of all personnel involved and building a rapport between a consistent recovery team and the implant center. In addition, the service combines this local surgical expertise with mEVLP technology, thus seeking to harness the potential advantages of ex situ perfusion in serial assessment of the donor organ, reconditioning, and increased tolerance to ischemia time. This combination of factors might encourage transplant teams to accept organs through a shared service when recovery would be logistically challenging for their own teams.

Sharing resources might be particularly advantageous in DCD. Previously identified barriers to DCD transplantation include logistics and the inability to predict progression to asystole accurately.^4^ These were the very reasons that prompted the use of the NOP service in this series. DCD recoveries have the greatest potential for a “negative run,” the mobilization of a recovery team without subsequent transplantation. This may occur with an incidence upward of 40% and is often attributed to an inability to predict progression to asystole in a reasonable time.^7^ DCD lung transplantation is presently associated with these resource commitments that limit utilization to centers with the means to support a DCD program. The variation between transplant programs in the adoption of DCD leads to a disparity between programs and diminishes equity within the transplant system. Adoption of local resources through a recovery service could go some way toward alleviating this disparity.

Our experience demonstrates the advantages of the NOP service, particularly in DCD, and the surgical expertise of the recovery team. The novelty of our described experience lies in the combination of a different-team procurement with EVLP technology, allowing challenging recoveries to be performed even when it would be logistically unfeasible or fiscally unfavorable for a program to send its own teams. However, widespread adoption of this service would require demonstration of equivalence in clinical outcomes and demonstration of cost-utility. Our limited experience to date demonstrates posttransplantation outcomes similar to those using standard recovery. Although this may go some way to alleviating concerns, more clinical data are required and will be forthcoming over time. Olaso and colleagues^8^ have recently demonstrated that different-team recoveries are profitable for institutions. This was particularly true with increasing interfacility distance. Unfortunately, the use of EVLP and DCD donors was limited in the study. We were unable, in this small series, to study the cost-utility of the service. Thus, the specific financial implications of using the NOP service require further investigation.

In conclusion, a novel national mEVLP recovery service is safe and effective even in challenging procurements. Such a program may be beneficial to increase organ utilization through expanded use of lung donors in general and DCD donors in particular. Further study is warranted for the impact on transplant program resources, organ utilization, and outcomes.
